# Metachronous descending colon volvulus after sigmoidectomy: a case report

**DOI:** 10.1093/jscr/rjae827

**Published:** 2025-01-03

**Authors:** Asratu G Amare, Gebrehiwot A Workneh, Mequanint T Tassew, Minale M Kebede, Mengist A Tegegne, Michael A Negussie

**Affiliations:** Department of Surgery, School of Medicine, College of Medicine and Health Sciences, University of Gondar, Maraki Street, Gondar City, Central Gondar Zone, P.O. Box 196, Gondar, Ethiopia; Department of Surgery, School of Medicine, College of Medicine and Health Sciences, University of Gondar, Maraki Street, Gondar City, Central Gondar Zone, P.O. Box 196, Gondar, Ethiopia; Department of Surgery, School of Medicine, College of Medicine and Health Sciences, University of Gondar, Maraki Street, Gondar City, Central Gondar Zone, P.O. Box 196, Gondar, Ethiopia; Department of Surgery, School of Medicine, College of Medicine and Health Sciences, University of Gondar, Maraki Street, Gondar City, Central Gondar Zone, P.O. Box 196, Gondar, Ethiopia; Department of Surgery, School of Medicine, College of Medicine and Health Sciences, University of Gondar, Maraki Street, Gondar City, Central Gondar Zone, P.O. Box 196, Gondar, Ethiopia; School of Medicine, College of Health Sciences, Addis Ababa University, Tikur Anbessa Specialized Hospital, Churchill Avenue, Lideta Sub-City, P.O. Box 5657, Addis Ababa, Ethiopia

**Keywords:** metachronous volvulus, sigmoidectomy, hemicolectomy, stoma, case report

## Abstract

Volvulus is the rotation or twisting of the intestine around its vascular pedicle. The occurrence of descending volvulus after sigmoidectomy is extremely rare. We report a case of a 35-year-old male who presented with abdominal distention, cramping, and no passage of feces or gas for three days. He had a history of recurrent sigmoid volvulus, previously treated with sigmoidectomy. On this occasion, clinical examination and imaging revealed a distended bowel with air-fluid levels. During exploratory laparotomy, descending colon volvulus, a rare finding, was confirmed. The patient underwent a left hemicolectomy and transverse stoma and recovered well postoperatively. Descending colon volvulus is a rare but serious complication after sigmoidectomy, and early diagnosis is essential. In volvulus-endemic regions, awareness of this condition is critical to prevent delayed diagnosis and complications.

## Introduction

Volvulus refers to an axial twist of the gastrointestinal tract, from the stomach to the rectum, along its mesentery [1]. This twisting can cause partial or complete bowel obstruction, leading to varying degrees of arterial and venous occlusion. The colon is the most common site of volvulus, with 65%–80% of cases involving the sigmoid colon, 43% involving cecum, 15%–30% involving the right colon, 2%–5% involving the transverse colon, and 2% involving splenic flexure [2, 3].

Untreated volvulus can result in bowel perforation, a life-threatening emergency. Common symptoms include abdominal pain and cramps, abdominal bloating, constipation, nausea, vomiting, and the inability to pass gas. Metachronous descending colon volvulus after a prior sigmoidectomy is an extremely rare occurrence. According to a literature review, very few cases of descending colon volvulus have been reported in patients with a history of sigmoidectomy [2].

This case report describes a 35-year-old male with descending colon volvulus following sigmoidectomy. Early recognition and timely intervention are crucial to prevent complications and improve outcomes.

## Case report

A 35-year-old male presented to the emergency department with a three-day history of abdominal distension, cramping abdominal pain, and inability to pass stool or gas. Ten years earlier, the patient had undergone a resection with primary end-to-end anastomosis for recurrent sigmoid volvulus. Intraoperative findings revealed a viable but redundant sigmoid colon, which was resected, followed by anastomosis. The patient recovered well but experienced recurrent abdominal discomfort, requiring rectal deflation on three occasions over the last 4 years.

**Figure 1:**
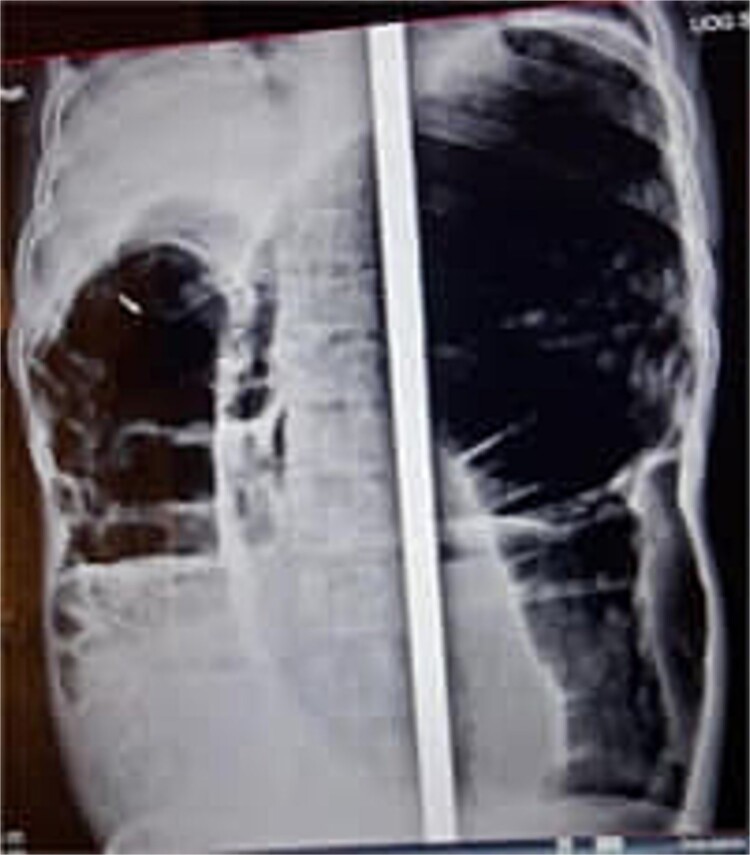
Abdominal X-ray showing dilated colon and multiple air-fluid levels.

At presentation, his vital signs were stable, but his abdomen was distended, and the rectum was empty upon digital examination. Blood tests revealed hypoalbuminemia (serum albumin of 2.2 g/dL), with normal electrolytes, complete blood count (CBC), and organ function tests. An erect abdominal X-ray showed distended bowel loops with air-fluid levels (Fig. 1). A differential diagnosis of sigmoid volvulus recurrence was considered. However, rectal decompression was unsuccessful. The patient’s condition worsened, with tachycardia (heart rate of 100 bpm), tachypnea (respiratory rate of 24 breaths per minute), fever (37.8°C), and abdominal tenderness. Repeat blood tests showed a left shift in the CBC, raising concerns about peritonitis.

**Figure 2:**
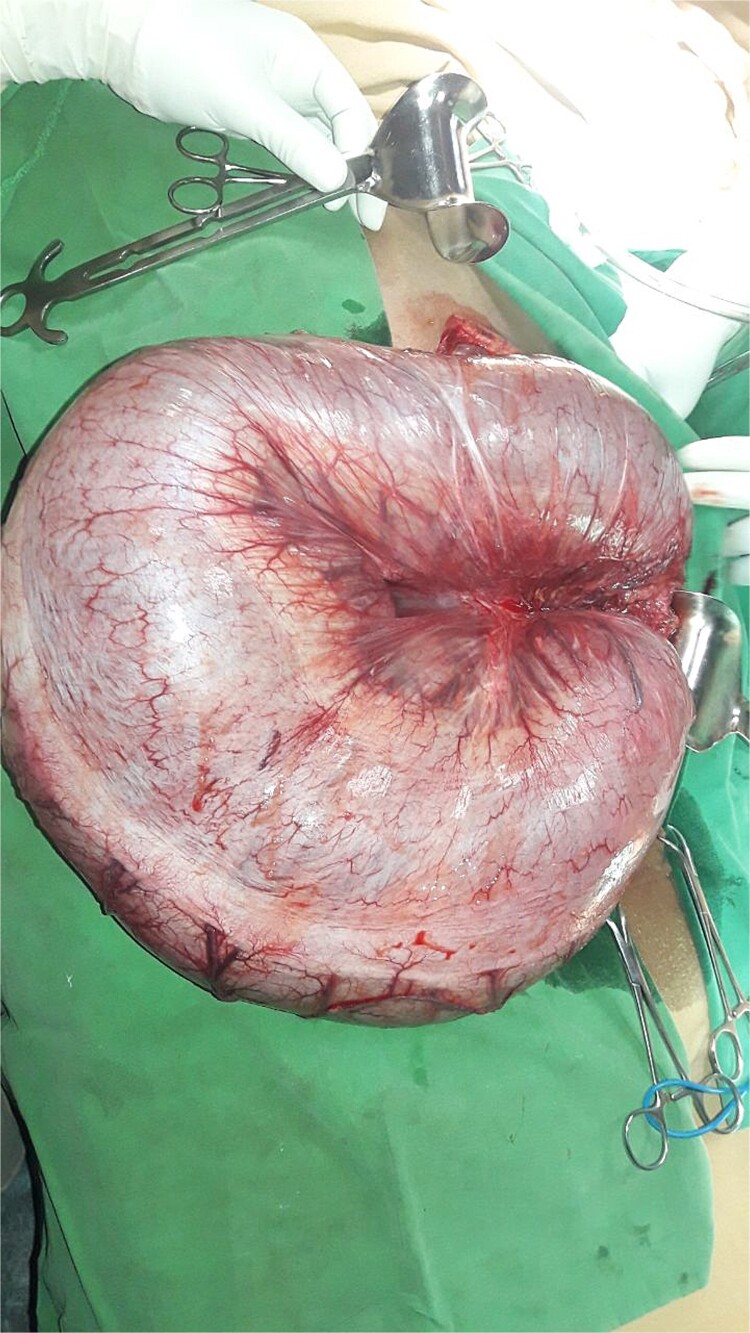
Photograph of descending colon volvulus.

**Figure 3:**
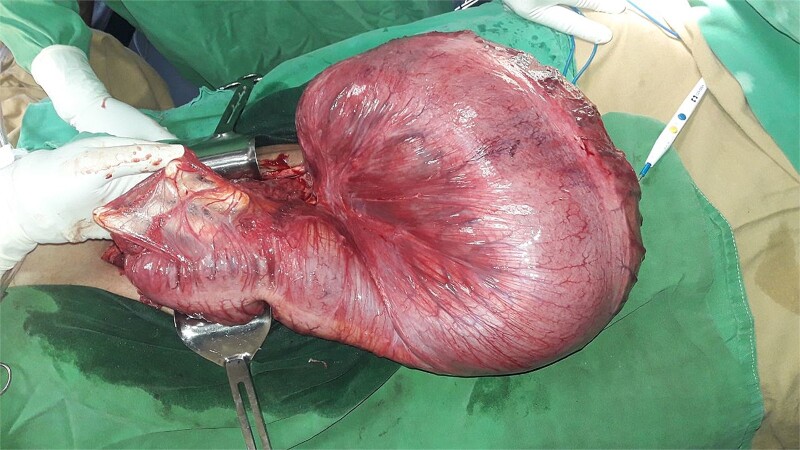
Photograph of the descending colon post-derotation.

After resuscitation, the patient underwent an emergency exploratory laparotomy. Intraoperative findings included a 360-degree counterclockwise rotation of the descending colon, with twists near the splenic flexure proximally and the peritoneal reflection distally. The descending colon was redundant with a narrow mesocolon. The entire colon was edematous and distended, with the affected bowel segment showing pronounced distension, edema, and dusky appearance, along with gangrenous areas at the twist sites (Figs. 2 and 3). A left hemicolectomy with transverse stoma creation was performed. The patient recovered well and was discharged on the fourth postoperative day. At his 10-day follow-up, the patient was tolerating oral feeds well and had no abdominal pain or distension. A follow-up 1-month post-surgery revealed that the patient remained stable with a functional stoma.

## Discussion

Colonic volvulus, characterized by the twisting of the bowel around its mesentery, is the third leading cause of colonic obstruction globally, following carcinoma and diverticulitis. The most common sites are the sigmoid and cecum, accounting for 60% and 34% of cases, respectively, with the transverse colon and splenic flexure being less frequently involved [4, 5]. Predisposing factors include a long, redundant colon, narrow mesentery, poor bowel habits, constipation, and surgical adhesions [4].

The incidence of colonic volvulus varies significantly by region. In the “volvulus belt” regions, including Africa, South America, Russia, Eastern Europe, the Middle East, India, and Brazil, volvulus accounts for 13%–42% of all intestinal obstructions. In contrast, it represents ˂5% of obstructions in Western countries. Sigmoid volvulus predominantly affects younger men in the volvulus belt, with a male-to-female ratio of 4:1. In Western countries, sigmoid volvulus typically affects older males (age >70), while cecal volvulus is more common in younger females (age ≤60) [6].

Descending colon volvulus is exceedingly rare due to the retroperitoneal location and lack of mesentery. However, failure of the primitive dorsal mesocolon to fuse with the parietal peritoneum during fetal development can result in a persistent descending mesocolon, increasing the risk of volvulus [7]. In this case, a preoperative diagnosis of descending colon volvulus was not made due to the unavailability of a computed tomography (CT) scan. Non-operative management, including colonoscopic decompression, is often the first-line treatment in uncomplicated cases, although recurrence rates are high, up to 85%. In patients who have undergone previous sigmoidectomy, the incidence of recurrent volvulus is reported to be between 6% and 36% [8, 9].

Management of colonic volvulus depends on the patient’s clinical status and the location of the volvulus. Surgical options include endoscopic detorsion, laparoscopic surgery, or resection of the affected bowel. If there are no signs of necrosis or perforation, colonoscopic decompression followed by elective resection is preferred, as it increases the likelihood of a one-stage definitive procedure. However, if detorsion fails or necrosis is suspected, surgery is necessary [10, 11].

A similar case of descending colon volvulus following sigmoidectomy was reported at Debre-Markos University, where the volvulus was derotated, and extraperitonealization of the descending colon was performed [9]. Another case from Mizan-Tepi University involved Hartmann’s resection following sigmoidectomy [12]. In both cases, non-operative management failed, underscoring the importance of surgery when rectal decompression is unsuccessful.

In volvulus belt regions, surgeons should remain vigilant for volvulus recurrence even after surgical intervention, as the risk remains significant. Early recognition and appropriate management are essential to minimize complications and improve outcomes [11, 12]. Familiarity with volvulus and its management in endemic regions has shown to improve patient outcomes [13].

## Conclusion

Metachronous descending colon volvulus after sigmoidectomy is a rare and challenging condition to diagnose. In volvulus belt regions, it should be considered in patients with a history of sigmoidectomy presenting with bowel obstruction. Early diagnosis and surgical intervention are critical to preventing severe complications. Even after successful surgery, careful monitoring for recurrence is necessary.
